# Prognostic factors and mitotane treatment of adrenocortical cancer. Two decades of experience from an institutional case series

**DOI:** 10.3389/fendo.2022.952418

**Published:** 2022-09-29

**Authors:** Judit Tőke, Andrea Uhlyarik, Júlia Lohinszky, Júlia Stark, Gergely Huszty, Tamás Micsik, Katalin Borka, Péter Reismann, János Horányi, Peter Igaz, Miklós Tóth

**Affiliations:** ^1^ Department of Internal Medicine and Oncology, European Reference Network on Rare Endocrine Conditions (ENDO-ERN) Health Care Provider (HCP), Faculty of Medicine, Semmelweis University, Budapest, Hungary; ^2^ Department of Internal Medicine and Haematology, Faculty of Medicine, Semmelweis University, Budapest, Hungary; ^3^ Department of Surgery, Transplantation and Gastroenterology, Faculty of Medicine, Semmelweis University, Budapest, Hungary; ^4^ First Department of Pathology and Experimental Cancer Research, Faculty of Medicine, Semmelweis University, Budapest, Hungary; ^5^ Department of Pathology, Forensic and Insurance Medicine, Faculty of Medicine, Semmelweis University, Budapest, Hungary; ^6^ Department of Endocrinology, European Network for the Study of Adrenal Tumours (ENS@T) Research Center of Excellence, Faculty of Medicine, Semmelweis University, Budapest, Hungary; ^7^ Magyar Tudományos Akadémia-Semmelweis Egyetem (MTA-SE) Molecular Medicine Research Group, Eötvös Loránd Research Network, Budapest, Hungary

**Keywords:** Adrenocortical cancer, prognostic markers, overall survival, mitotane, single centre experience

## Abstract

**Objectives:**

This study aimed to characterise the clinicopathological features and prognostic factors of a large cohort of Hungarian patients with adrenocortical cancer diagnosed between 2000-2021.

**Patients and methods:**

This retrospective study included seventy-four patients (27 men and 47 women) with histologically confirmed adrenocortical cancer in a single tertiary referral endocrine centre. Descriptive statistics were performed, providing summaries of selected clinical and pathological parameters. Clinicopathological factors contributing to overall survival were analysed.

**Results:**

The median age of patients was 48,5 years (17-84 years) at diagnosis. The majority of cases were diagnosed at ENSAT stage II (39,2%) and stage IV (33,8%). At diagnosis, the median tumour size was 9,0 cm (4,5-20 cm). In 47 patients (71,6%), the tumour was hormonally active. The median overall survival and the 5-year survival rate were 23,5 months (95% CI, 17-30,5 months) and 18,3%, respectively. Primary tumour resection was performed in 68 patients (91,8%); R0 surgical resection was achieved in 30 patients. In univariate Cox regression model, tumours with stages III and IV, high proliferative activity (Ki67-index > 10%), R1-R2 surgical resection state and hormonal activity were associated with poorer survival. Cortisol excess, both isolated and combined with androgen production, was associated with poorer survival. Fifty-five patients were treated with mitotane. The overall survival of patients achieving therapeutic mitotane plasma concentration was significantly better compared to those who never reached it [27.0 (2-175) months vs 18.0 (2-83) months; p<0.05)]. The median age, the distribution of gender, ENSAT stage, resection state and Ki67-index did not differ between these two groups. The time needed to reach the therapeutic range of serum mitotane was 96.5 days (95% CI, 75-133 days).

**Conclusion:**

Our results confirm previous data that disease stage, mitotic activity, the resection state and the mitotane treatment achieving therapeutic concentration are the most critical parameters influencing the prognosis of adrenocortical cancer. Our data suggest that hormonal activity may be more frequent than described previously, and it is a strong and independent prognostic factor of overall survival. To our knowledge, this is the first single-centre study confirming the prognostic importance of achieving therapeutic mitotane concentration.

## Introduction

Adrenocortical cancer (ACC) is a rare malignant tumor usually with aggressive biological behaviour and poor prognosis. The incidence is 0.7-2.0/million/year. The only potentially curative treatment is surgical resection of the primary tumor with regional lymph node dissection (1). However, more than half of stage I-III ACC patients relapse after adrenalectomy. Concerning patients with advanced disease, removal of distant metastases could provide prolonged survival only in properly selected patients with oligometastatic ACC (2). To date, mitotane is the only drug approved for the treatment of ACC. Mitotane can be administered in an adjuvant setting, while in advanced disease stages, it is the standard treatment either in monotherapy or in combination with cytostatic chemotherapy (3, 4). Despite our continuously deepening knowledge regarding tumor biology of adrenocortical cancer, our therapeutic armamentarium remained unchanged in the past decades, and the median overall survival of patients with ACC is still around 2-4 years (5–7).

To date, only a few single-centre studies published overall survival data in patients with advanced ACC as a function of mitotane treatment. In 2013, Ayala-Ramirez et al. from The University of Texas MD Anderson Cancer Center demonstrated a clear difference in overall survival between patients with ACC who reached and who failed to reach 14 mg/L mitotane plasma concentration [4.1 years (95% CI: 2.8-7.0) vs 2.9 years (95% CI: 2.2.-3.8 years)] (5). In a smaller cohort of Serbian patients, Loncar et al. confirmed with univariate Cox-regression analysis that mitotane treatment was associated with favourable overall survival (HR to death: 0.13, 95% CI: 0.06-0.31) (8).

This study aimed to characterise the clinicopathological features and prognostic factors of a large cohort of patients with adrenocortical cancer diagnosed and/or treated in Hungary’s largest tertiary referral centre for adrenal disorders. Furthermore, we aimed to assess the impact of mitotane treatment on patient outcomes.

## Patients and methods

This retrospective study included adult patients with histologically confirmed adrenocortical cancer consecutively diagnosed and/or treated in our unit between 2000 and 2021.

Routine endocrinological testing included measurements of serum cortisol (circadian rhythm and after low dose dexamethasone suppression), testosterone, dehydroepiandrosterone sulfate, 17-hydroxy-progesterone and plasma ACTH. All laboratory results were reevaluated for the proper classification of hormonal activity of adrenocortical cancers.

For those patients who were operated on outside of our centre (n=28), the hormonal activity of the tumors was determined using the results of the referring endocrine units. In cases with residual tumor or tumor recurrence, endocrine testing was repeated in our centre.

In addition, plasma aldosterone and plasma renin activity were measured in hypertensive patients. Occasionally, other steroid hormones (mineralocorticoid precursors, oestradiol, etc.) were also examined. Multiple hormone secretion was diagnosed if more than one adrenal hormone production was confirmed with laboratory testing.

Descriptive statistics were used to characterise selected clinical and pathological parameters at diagnosis. The tumor stage was determined according to the staging system proposed by the European Network for Study of Adrenal Tumors (ENS@T) (9). The absence or presence of residual tumor following surgical resection was categorised as suggested by the American Joint Committee on Cancer (10). Rx reflects that the presence of a residual tumor can not be assessed. R0 represents even microscopic lack of residual tumor. R1 and R2 reflect microscopic and macroscopic residual tumor at the primary cancer site, respectively.

During the two decades of this retrospective study, mitotane was used mainly in ACC patients with stage IV disorder but not in an adjuvant setting. For mitotane treatment, we used Lysodren^®^ 500 mg tablets. During the titrating phase of mitotane treatment, our primary aim was to rapidly increase the daily mitotane dose (starting dose 1000-1500 mg; followed by 500-1000 mg daily increment until 6000 mg). After that, the dose was adjusted based on plasma mitotane concentration, aiming to reach the mitotane therapeutic range between 14-20 mg/L. The timing for assessment of plasma mitotane concentration and dose determination were individually decided by the responsible physician. The measurement of plasma mitotane concentrations was provided by HRA Pharma. Blood samples were collected in our centre and sent to the Lysosafe service laboratory, where plasma mitotane concentrations were measured using a standardised gas chromatography/mass spectrometry.

For categorical variables, counts and percentages were calculated. Continuous data are presented as medians and ranges (minimum-maximum). Proportions of categorical variables were compared by chi-squared test or Fisher’s exact test as appropriate, while differences in continuous variables were analysed by the Mann–Whitney U test. The survival curves were obtained from the Kaplan–Meyer analyses followed by log-rank tests. Factors contributing to overall survival were analysed with univariate Cox proportional hazard model. All variables associated with increased risk of death at a p < 0.05 level of significance were included in the multivariate Cox regression analysis. A p-value of less than 0.05 was considered statistically significant. All statistical analysis was performed with IBM SPSS Statistics for Windows, Version 27.0. Armonk, NY: IBM Corp.

The study was approved by the local ethical committee of Semmelweis University (TUKEB 38/2022). The patients’ identification was kept confidential. Due to the retrospective design, informed consent was not required.

## Results

This retrospective study included 74 adult patients, 27 men and 47 women with histologically confirmed adrenocortical cancer. At the time of diagnosis, the median age was 48 years (range: 20-79 years) for men and 51.0 years (range: 17-84 years) for women. Fifty patients have been diagnosed and treated since 2010.

The essential demographic and tumor characteristics of our patients diagnosed with adrenocortical cancer are summarised in **Table 1**. The median diameter of the tumors was 9.0 cm (4.5-20 cm). Seventy-one patients were categorised according to the ENSAT staging classification. Thirty-one (43.7%) patients presented with tumors confined to the adrenals (two and twenty-nine patients for ENSAT stages I and II, respectively.) Our two patients with stage I disease were discovered incidentally. Fifteen and twenty-five patients were diagnosed with stages III and IV, respectively. Most of the patients (71.6%) presented with clinical and/or biochemical evidence of hormone excess. Among the hormonally active tumors, combined cortisol and androgen production was diagnosed most frequently, followed by isolated cortisol- and isolated androgen secretion (46.8%, 38.3% and 10.7%, resp.).

**Table 1:** Descriptive statistics of our ACC patients’ cohort (n = 74).

Gender	n (%)
Male	27 (36.5%)
Female	47 (63.5%)
**Age at time of diagnosis**	**years (median; min-max)**
Male	48.0 (20-79)
Female	51.0 (17-84)
**Maximum tumour diameter (median, min-max)**	
	9.0 (4.5 – 20) cm
**ENSAT classification 2010 (n = 71)**	
Stage I	2 (2.8%)
Stage II	29 (40.9%)
Stage III	15 (21.1%)
Stage IV	25 (35.2%)
**Resection state (n = 55)**	
Rx	10 (18.2%)
R0	30 (54.5%)
R1	13 (23.6%)
R2	2 (3.6%)
**Recurrence rate in patients with ENSAT I-III stages (n = 46)**	
Locoregional recurrence	21 (45.6%)
Distant metastases	27 (58.7%)
**Hormonal activity (n = 68)**	
Nonfunctioning tumour	21 (28.4%)
Functioning tumour	47 (71.6%)
◼ Multiple hormone secretion	◼ 22 (46.8%)
◼ Cortisol	◼ 18 (38.3%)
◼ Androgenes (testosterone, DHEAS)	◼ 5 (10.7%)
◼ Aldosterone	◼ 1 (2.1%)
◼ Oestradiol	◼ 1 (2.1%)
**Patients with diabetes mellitus (n = 16)**	
◼ Number of patients with cortisol excess	◼ 14 (87.5%)
◼ Number of patients without cortisol excess	◼ 2 (12.5%)
**Treatment**	
Surgical removal of the primary tumour	68 (91.8%)
Tumour bed irradiation	16 (21.6%)
Mitotane treatment	55 (74.3%)
◼ Monotherapy	◼ 17 (30.9%)
◼ Combined with platinum-based chemotherapy	◼ 38 (69.1%)
First-line chemotherapy	38 (51.4%)
◼ EDP protocol	◼ 21 (55.3%)
◼ other platinum-based chemotherapy	◼ 14 (36.8%)
◼ cyclophosphamide	◼ 2 (5.3%)
◼ gemcitabine + capecitabine	◼ 1 (2.6%)

ENSAT, European Network for the Study of Adrenal Tumors; DHEAS, Dehydroepiandrosterone sulfate; EDP, Etoposide-Doxorubicin-Cysplatin.

Adrenalectomy was performed in 68 patients (91.8%). Four of the six unoperated patients died close to the establishment of the clinical diagnosis, while 2 patients refused the surgical intervention. The primary tumor proved to be unresectable in three cases. The primary tumor proved to be unresectable in three cases. All patients with tumors confined to the adrenal region were operated on (n=46), and even 19 of 25 patients with advanced, metastatic disease underwent primary tumor resection. In the latter subgroup, those who had cytoreductive adrenalectomy (n=19) exhibited significantly longer survival compared to those who was not operated (n=6) (14.0 (2.0-177.0) months vs. 2 (0.0-12.0) months, p=0.002). The overall survival of patients with laparoscopy vs laparotomy has not significantly differed (42.0, 95% CI: 6.3-77.6 months for patients with laparoscopy vs 26.0, 95% CI: 21.1-30.8 months for those with laparotomy, p=0.365).

Forty patients were operated on at our institution by high-volume adrenal surgeons, while 28 patients had primary tumor resection in other surgical units. Tumor-free surgical margins (R0 resection) were achieved in 30 patients (44.1% of all operated cases). Complete tumor resection (R0) was significantly more frequent in patients who were operated in our institution [68.8% (22/32) vs. 33.3% (7/21), p=0.011]. However the overall survival did not differ between patients operated in our institution vs outside (33.0 months, 95% CI: 16.0-49.9 months, vs 26.0 months, 95% CI: 12.3 - 39.6 months, resp., p=0.745).

Fifty-five patients were administered therapeutic mitotane. The median treatment period was 15 months (CI 95%, 10-19 months). The indication for mitotane treatment was the presence of residual tumor or tumor recurrence and metastatic stage IV disease. First-line mitotane monotherapy was administered in 17 patients, while mitotane combined with platinum-based chemotherapy (most frequently etoposide-doxorubicin-cisplatin – EDP) was used in 38 patients in the first line. Chemotherapy was indicated in fit enough patients with surgically untreatable, progressive metastatic disease.

In the 46 patients operated with ENSAT I-III stages, locoregional recurrence and distant metastases developed in 21 (45.6%) and 27 patients (58.7%), resp. The median time to locoregional recurrence and distant metastases were 7 months (range: 0-97 months) and 9 months (range: 0-97 months), resp. Postoperative tumor bed irradiation was performed in 16 cases (21.6% of all and 34.7% of ENSATI-III patients).

Survival analysis was performed using the date of death in the case of the deceased (n=52) and the date of the last contact in the case of lost-to-follow-up patients as censored (n=22). Of the 74 patients, 66 patients were followed until death (n=52) or last contact (n=14). The median follow-up time was 22 months (CI 95%, 16-31). The median overall survival time and the 5-year survival rate were 23.5 months (95% CI, 17-30.5 months) and 18.3%, respectively. Four patients had died before any cancer-specific treatment (including even surgical intervention) could be given due to a lethal complication of Cushing’s syndrome (thromboembolic events, opportunistic infections and fatal hepatic failure). After excluding these four patients, the median overall survival and the 5-year survival rate were 24.0 months and 20%, respectively.

The relationship between clinical parameters and mortality obtained from the univariate Cox proportional hazard model is presented in **Table 2**. Tumors with stage III-IV, high proliferative activity (Ki67-index > 10%), R1-R2 surgical resection and inability to reach therapeutic plasma mitotane concentration were associated with an increased risk of death. The range of the Ki67-index was between 4 and 70%. We assessed the prognostic value of the Ki67-index with various cut-offs. The best cut-off for Ki67 differentiating patients according to their overall survival was 10%. Cortisol excess, either isolated or combined with androgen secretion, was associated with an elevated risk of death (RR 2.469 [95% CI: 1.09-5.59] and RR 3.425 [95% CI: 1.549-7.574], resp.). Diabetes mellitus was also associated with an increased risk of death (RR 2.184.[95% CI: 1.079-4.420]. It should be noticed that 14 out of the 16 patients with diabetes also had cortisol-producing tumors. Gender, age at diagnosis, tumor size, Weiss score and the time needed to reach therapeutic mitotane concentration were also analysed. These factors, however, did not influence survival.

**Table 2:** Factors associated with mortality of patients with adrenocortical cancer using univariate Cox proportional hazard model.

		**RR of death**	**95% C. I.**	**p value**
**Gender**				
	Male	1.0		
	Female	1.482	0.831 – 2.643	0.183
**ENSAT stage**				
	stage I	–	–	–
	stage II	1.0		
	stage III	1.738	0.844-3.579	0.134
	stage IV	3.341	1.735-6.432	< 0.001
**ENSAT stage**				
	stage I and II	1.0		
	stage III and IV	2.68	1.507-4.767	< 0.001
**Resection state**				
	R0	1.0		
	R1 or R2	2.567	1.174-5.614	0.018
**Ki67-index (%)**				
	< 10	1.0		
	≥ 10	3.897	1.143-13.288	0.030
**Hormonal activity**				
	No	1.0		
	Yes	3.005	1.480-6.105	0.002
**Hormonal activity**				
	non-functioning	1.0		
	cortisol	2.469	1.090-5.592	0.030
	androgens	1.949	0.598-6.351	0.268
	cortisol+androgenes	3.425	1.549-7.574	0.002
**Diabetes mellitus at diagnosis**				
	No			
	Yes	2.184	1.079-4.420	0.030
**Mitotane therapeutic range reached**				
	Yes	1.0		
	No	2.012	1.081-3.746	0.027
				

C.I., Confidential Interval; RR, relative risk.

All variables associated with increased risk of death at a p < 0.05 level of significance were included in the multivariate Cox regression analysis. The hormonal activity was the only one that proved to be an independent prognostic factor (**Table 3** **).**

**Table 3:** Multivariate Cox regression analysis of clinical parameters associated with mortality in univariate analysis.

Variable	RR of death	95% C. I.	p value
ENSAT stage (III-IV vs. I-II)	3.575	0.594-21.505	0.164
Resection state (R1 or R2 vs. R0)	0.789	0.060-10.332	0.857
Ki67-index (≥ 10% vs. < 10%)	9.294	0.789-109.432	0.076
Mitotane therapeutic range (reached vs. not reached)	0.859	0.191-3.874	0.843
Hormonal activity (active vs. inactive tumours)	8.389	1.274-55.220	0.027
Diabetes mellitus (present vs. absent at diagnosis)	1.775	0.331-9.524	0.503

**Figure 1:**
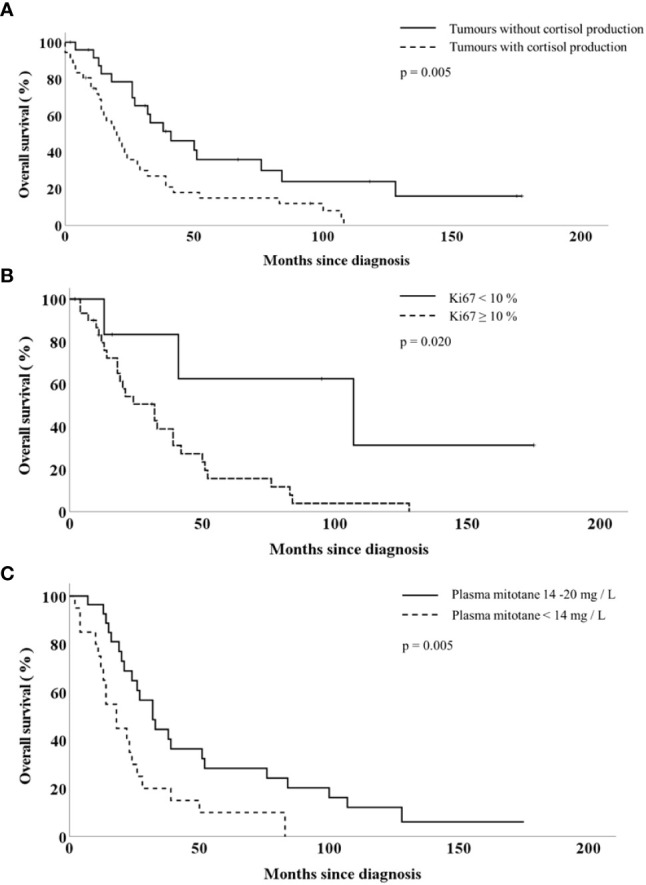
Overall survival of patients with adrenocortical cancer according to the tumour cortisol production **(A)**, tumour Ki67-index **(B)** and the plasma mitotane concentration **(C)**.

Out of the 55 patients treated with long-term mitotane therapy, therapeutic plasma mitotane concentration could be achieved in 29 patients. The median time needed to reach the therapeutic range of plasma mitotane concentration was 96.5 days (95% CI, 75-133 days)/3 months (95% CI, 2-4 months)) with a median of 4.0 (CI 95%, 4.0-5.5) measurements of plasma mitotane concentration. Mitotane-treated patients having survived more than 12 and more than 24 months were sampled for plasma mitotane measurement an average of eight times (CI 95%, 3.0-14.0) and of two times (CI 95%, 2.0-4.0) during the first and second years of treatment, resp. The overall survival of patients achieving therapeutic mitotane plasma concentration was significantly longer compared to those who failed to achieve it [(27.0 (2–175) months vs 18.0 (2–83) months, p<0.05)]. Apart from plasma mitotane concentrations, there were no statistically significant differences in other clinical or prognostic parameters (age, gender, resection state, Ki67-index, surgical intervention, hormonal activity) between these two groups of patients (**Table 4** **)**. The survival curves obtained by Kaplan-Meyer analyses are shown in **Figure 1**.

**Table 4:** Clinicopathological characteristics of patients who reached vs never reached therapeutic mitotane range.

	**Mitotane therapeutic range never reached (n = 21)**	**Mitotane therapeutic range reached (n = 29)**	**p**
Median overall survival (mo)	18 (2-83)	27 (2-175)	0.037
			
Age (years, median, min-max)			
	47.0 (19-72)	49.0 (17-79)	NS *
Male/Female			
	5/16	12/17	NS **
Resection stage			
R0	6	12	NS **
R1 or R2	4	9	
Ki67-index (%, median, min-max)			
	25 (5 – 52)	15 (4 – 70)	NS *
Number of patients with adrenalectomy			
	19	29	NS **
Number of patients with R0 resection			
	6	12	NS**
Number of patients with chemotherapy			
	14	20	NS **
Number of patients with hormonally active tumour			
	16	17	NS **
Number of patients with cortisol secreting tumour			
	4	7	NS **
Number of patients with multiple hormone-secreting tumour			
	7	8	NS **

NS, non-significant; * Mann-Whitney U-test; ** Chi-squared test.

## Discussion

Managing patients with rare diseases is always challenging, as no high-level evidence is available. The ESE-ENS@T clinical practice guideline on the management of adrenocortical carcinoma emphasises the benefit of patients’ registries to collect and analyse clinicopathological data to improve the level of evidence of diagnostic accuracy and treatment efficacy (11).

This retrospective analysis of our institutional registry was conducted to evaluate clinicopathological features of patients with adrenocortical cancer. The study also included survival analyses and investigations of known or presumed clinical factors and prognostic parameters, including the impact of mitotane treatment, determining overall survival.

Regarding demographics, our study cohort is comparable to previously published case series. However, the proportion (71.6%) of patients with hormonally active ACC is higher in our series than in most other publications (5, 8, 12–15) (**Table 5**). For example, in a recent meta-analysis with 3814 cases, 50,4% of all patients had hormonally active ACC (17). The different rate of hormonally active tumors probably reflects differences in the local diagnostic protocols and laboratory methods used for the endocrine investigations of ACC patients. The introduction of LC/MS method for hormonal measurements led to an increase in the detection of the rate of hormonally active adrenal tumors (16, 18). However, it is worth noting, that the rate of hormonally active tumors in our cohort was approximately as high as in a Swedish ACC cohort tested by urinary steroid profiling (16). Since our institution introduced LC/MS only in 2019, we have not got enough experience with this method in ACC patients (19). The proportion of patients with hormonally active ACCs may also vary depending on the main profile of the referral centre, endocrinology or oncology.

**Table 5:** Prognostic factors and survival in single-centre series with adrenocortical carcinoma.

Author (year)	No of patients(M/F)	Proportion of hormonally active tumours (%)	ENSAT stage I-II-III-IV (%), resp.	5-year survival rate (%)	Overall survival (median months, 95% CI or mean ± SEM months)	Factors associated with overall survival	(Hazard ratio, 95% CI)	Factors *not* associated with overall survival
Ayala-Ramirez M et al, (2013) (5),	330 (M:118/F:212)	41.8%	I: 3.3% II: 37.3% III: 33.6% IV: 25.8%	38%	38.4 mo (32.4–48.0)	*multivariate Cox regression:* older age functioning tumor ENSAT I or II	(HR: 1.013, 1.003–1.024) (HR: 1.400, 1.055–1.857) (HR: 0.438, 0.325–0.590)	gender resection state venous thromboembolism resection of >5 lymphnode
Else T et al, (2014) (13)	391 (M:158/F:233)	57.0%	I: 3.0% II: 43.0% III: 28.0% IV: 26.0%	ENSAT I: 40.4% ENSAT II: 58.0% ENSAT III: 24.5% ENSAT IV: 5.7%	35.2 mo (28.7– 41.6)	*univariate Cox regression:* older age cortisol production ENSAT III ENSAT IV High grade (≥ 20 mitoses per 50 hpf)	(HR: 1.011, 1.002–1.020) (HR: 1.422, 1.107–1.827) (HR: 2.192, 1.598 –3.006) (HR: 4.820, 3.535– 6.573) (HR: 2.535, 1.843–3.485)	gender localizsation (right vs left) androgen production
Loncar Z et al, (2015) (8)	72 (M:30/F: 42)	26.4%	I: 3.1% II: 48.4% III: 31.3% IV: 17.2%	41.1%	36.0 mo (13.4–58.5)	*univariate Cox regression:* male gender age >50 ys at diagnosis ENSAT III or IV tumour weight > 300gr extraperit. surgical approach mitotane used	(HR: 2.170, 1.090–4.320) (HR: 2.400, 1.180–4.860) (HR: 2.940, 1.390–6.210) (HR: 2.260, 1,010-5,050) (HR: 0,290, 0,100-0,840) (HR: 0.130, 0.060–0.310)	localisation (right vs left) tumour size type of surgery reoperation surgeons experience hormonal activity
Calissendorff J et al, (2016) (16)	50 (M: 24/F: 26)	82.0%	I: 0% II: 50% III: 16% IV: 30%	40.0%	5.5 years (0.3–19.8)	*Kaplan-Meier analysis:* R0 vs. R1 resection R1 vs. R2 resection ENSAT III vs. ENSAT IV.		age > 40 years at diagnosis Ki67-index (> 10%) abnormal urine steroid profile
Punjani N et al, (2018) (14)	29 (M:14/F:15)	41.0%	I: 0% II: 28.0% III: 41.0% IV: 31.0%	27%	ND.	ND.		ND.
Bronswijk MJH et al, (2020) (12)	49 (M:17/F:32)	29.0%	I: 15.6% II: 28.9% III: 33.3% IV: 22.2%	48% (estimated)	45.6 mo (ND)	*univariate Cox regression:* higher ENSAT stage R1 or R2 resection metastasis at diagnosis	(HR: 2.989, ND) (HR: 3.883, ND) (HR: 2.326, ND)	surgery at relapse hormonal excess nodal involvement use of chemotherapy time to progression
Parianos C et al, (2021) (15)	45 (M:19/F:26)	37.7%	I: 6.6% II: 62.3% III: 26.7% IV: 4.4%	18.4%	ENSAT I-II: 62 ± 8.7 mo ENSAT III-IV: 31 ± 12.1 mo	*multivariate Cox regression:* tumour volume > 400 cm3 Weiss-score > 5 p53 overexpression	(HR: 11.472, 1.046-125.799) (HR: 212.502, 4.410-10240.462) (HR: 21.287, 1.214-373.182)	male gender, age > 50 ys ENSAT III or IV
Tőke J et al, (2022)	74 (M: 27/F:47)	71.6%	I: 2.7% II: 39.2% III: 20.3% IV: 33.8%	ENSAT I: 50.0% ENSAT II: 24.1% ENSAT III: 20.0% ENSAT IV: 4.0% all: 20.0% *	ENSAT I-II: 38.5 mo (23-50.5) 45.7 ± 7.5 mo ENSAT III-IV:19.5 mo (14-26) 26.6 ± 4.9 mo All: 24.0 mo (18-35.5)*	*univariate Cox regression:* ENSAT III or IV Ki67-index > 10% functioning tumor diabetes at diagnosis mitotane TR not reached R1 or R2 resection	(HR: 2.68, 1.507-4.767) (HR: 3.897, 1.143-13.288) (HR: 3.005, 1.480-6.105) (HR: 2.184, 1.079-4.420) (HR: 2.012, 1.081-3.746) (HR: 2.567, 1.174-5.614)	male gender age at diagnosis tumour size Weiss-score time to reach mitotane TR

*:Patients whose survival was less than 4 months from the diagnosis without any specific treatment (n = 4) have been excluded from the analyses.

M, male; F, female; ENSAT, European Network for the Study of Adrenal Tumors; mo, months; ys, years; TR, therapeutic range; EDP, Etoposide-Doxorubicin-Cisplatin; ND, no data..

Regarding the potential prognostic parameters of ACC, using the univariate Cox regression model, we have confirmed that tumors with stage III-IV, high proliferative activity, R1-R2 surgical resection state and inability to reach therapeutic plasma mitotane concentration were associated with poorer overall survival. In our series, Ki67 was found to have prognostic significance at a cut-off of 10%, while in other series, this value varies between 4 and 20% (15, 20, 21). However, in the multivariate analysis, Ki67 was found to have only borderline significance, probably due to a lack of power related to the size of our cohort. Furthermore, we have demonstrated that hormonal activity, more precisely cortisol production, is a strong and independent predictor of worse clinical outcomes. This observation is not surprising because Cushing syndrome of any origin significantly accounts for elevated risk of morbidity and mortality (22–24). The negative prognostic impact ofd hormonal activity (most probably of cortisol overproduction) is underlined by the fact that 4 out of 74 patients died due to endocrine complications but not tumoral progression.

However, to date, only a few single-centre studies proved that hormonal excess increases the mortality of patients with ACC (5, 13). Our institutional experience demonstrates that cortisol overproduction, either isolated or combined with androgens, is associated with an increased risk of death. Furthermore, our assessment of the effect of androgen secretion confirmed previous data from a US single-centre that isolated androgen production is not associated with overall survival (13).

Regarding the potential prognostic role of diabetes mellitus found in the univariate model, we have to underline that all but two patients with diabetes mellitus had cortisol-producing tumors. However, in the multivariate regression analysis, diabetes has lost significance, and hormonal activity remained the only independent risk factor.

When comparing 5-year survival rates and median overall survival data obtained from institutional case series, it should be noted that there is a high variance in the prevalence of two outstanding prognostic parameters, i. e. the stage and the hormonal activity of ACC. When comparing to other series, the proportions of patients with hormonally active tumor and stage IV disease were the highest in our series (**Table 5**).

Regarding treatment modalities, our practice is in line with current guidelines as most of our patients underwent adrenalectomy, even those with metastatic stage IV disease. Until recently, we lacked reliable survival data of patients with stage IV ACC regarding the impact of removing the primary tumor. In 2021, Srougi et al. summarised the survival results in a large American-Australian-Asian cohort of patients with metastatic ACC. They demonstrated that surgical removal of the primary tumor was associated with better survival even in stage IV disease (25). Our practice aligns with their recommendation, as two-thirds of our patients with stage IV disease underwent resection of the primary tumor. The indication of adrenalectomy in this subset of patients was decided on an individual basis at multidisciplinary tumor board meetings. Cytoreductive adrenalectomy was suggested for those stage IV patients with good performance status and in whom the visually estimated adrenal tumor/total body tumor volumen was > 80%.

Several studies reported that patients’ surgical outcome (proportion of R0 resection, locoregional recurrence rate, etc.) is better in specialised, large centres where the surgeon’s volume is high, and an adequate multidisciplinary approach is available (26, 27). As resection state after ACC surgery is a significant predictor of survival; based on our own experience and in accordance with current guidelines, we suggest that surgical treatment of ACCs should be carried out in centres with specific experience in adrenal surgery (11, 28, 29).

Mitotane was used in 74.3% of all cases. To date, no clinical or pathological marker is known that can reliably predict the response to mitotane. The first report on the association between mitotane use in the adjuvant setting and therapeutic outcomes was published by Terzolo et al. (30). This study reported survival data of patients from six European referral centres receiving mitotane after radical resection of ACC. Those patients who achieved and maintained ≥14 mg/L plasma concentration of mitotane exhibited longer recurrence-free survival, although the risk of death was not significantly altered (30).

The clinical practice regarding the starting dose and pace of dose-escalation of mitotane is highly variable; low- and high-dose approaches are used (11). Puglisi et al. reported that the time needed to achieve the target range of plasma mitotane and the time in the target range (14-20 mg/L) might influence treatment outcome. The authors of these multicentric analyses presented survival data of Italian ACC patients treated between 2005 and 2017. Multivariate analysis showed that the time required to achieve the target range of plasma mitotane was an independent predictor of recurrence-free survival. Similarly, a longer time in the target range of plasma mitotane was associated with a significantly lower recurrence risk and with prolonged overall survival (4, 31). In this context, it is interesting to note that the dosing regimen used in our centre resulted in a shorter time needed to achieve the mitotane target range as compared to the multicentric Italian study (31) [3 months (95% CI, 2-4 months) vs 8 months (IQR 5–19)]. However, in a prospective trial conducted with ACC patients with ENSAT III or IV stage, Kerkhofs et al. demonstrated that even 46-55 days could be enough to reach therapeutic mitotane concentration (32).

According to our best knowledge, our present analysis is the first single-centre report which could demonstrate that achieving therapeutic mitotane plasma concentration is associated with prolonged overall survival.

The main limitation of our study is its retrospective nature, which is partly counterbalanced by our efforts with a prospectively led institutional database. About one-fourth of survival data are censored since the exact date of death remains unknown. In addition, the primary profile of our centre, which is endocrinology, may have caused referral bias. The relatively high number of primary surgical interventions outside our institution did not allow us to systematically conduct hormonal investigations according to our diagnostic protocols. On the other hand, it allowed us to compare the success rate in surgical resection in low vs high- volume centres. Considering our study’s retrospective design, the cohort’s size, and the continuously evolving and changing laboratory methods of hormone measurements, it seems impossible to separately study the survival impacts of oncological interventions and endocrinological treatments aiming to reduce cortisol excess. These facts underline and strengthen the need for prospective, well-designed, systematically conducted multicentric registries for patients with ACC.

In conclusion, results from our institutional registry confirm previous data that disease stage, mitotic activity, resection stage and achievement of therapeutic mitotane concentration are the most critical factors influencing the prognosis of patients with adrenocortical cancer. According to our data, the prevalence of hormonal activity among patients with ACC may be as high as 70%. Hormonal activity proved to be the strongest predictor of poorer clinical outcomes.

## Data availability statement

The dataset analyzed during the current study is available from the corresponding author upon reasonable request.

## Ethics statement

The studies involving human participants were reviewed and approved by Semmelweis University Regional and Institutional Committee of Science and Research Ethics. Written informed consent for participation was not required for this study in accordance with the national legislation and the institutional requirements.

## Author contributions

JT and MT wrote the first draft of the manuscript. JT, AU, JL, GH, TM, KB, PR, JH, PI and MT participated in the diagnosis and treatment of the patients, provided follow-up. JT, PR and JS acquired clinical data. All authors contributed to the article and approved the final manuscript.

## Conflict of Interest

The authors declare that the research was conducted in the absence of any commercial or financial relationships that could be construed as a potential conflict of interest.

## Publisher’s note

All claims expressed in this article are solely those of the authors and do not necessarily represent those of their affiliated organizations, or those of the publisher, the editors and the reviewers. Any product that may be evaluated in this article, or claim that may be made by its manufacturer, is not guaranteed or endorsed by the publisher.
